# DSS Colitis Model: Traps, Tricks, and Reporting Recommendations

**DOI:** 10.3390/biomedicines14040928

**Published:** 2026-04-18

**Authors:** Martina Perše

**Affiliations:** Medical Experimental Centre, Institute of Pathology, Faculty of Medicine, University of Ljubljana, 1000 Ljubljana, Slovenia; martina.perse@mf.uni-lj.si

**Keywords:** DSS, colitis, visceral pain, gut–brain axis, gut–liver axis, reporting, genetic, gut microbiota, immune cells, cytokines, inflammation, behaviour

## Abstract

The dextran sodium sulfate (DSS) colitis model is the most widely used experimental model of inflammatory bowel disease (IBD) due to its simplicity and versatility, with over 7000 PubMed entries in the last decade and an exponential rise in recent years. Since its initial description in 1985, DSS colitis has been extensively evaluated across species, most notably in mice and rats, and has yielded substantial insights into IBD pathogenesis. However, the model’s multifactorial nature poses a dual challenge: it offers an opportunity but complicates study design, interpretation, and translational relevance. This complexity is worsened by inconsistent reporting, which hampers reproducibility and comparability across studies. The broad use of the DSS-induced colitis model yields numerous insights about the model, which help better understand its complexity, characteristics and limitations. Although DSS colitis is induced locally, inflammation in the colon and gut barrier destruction may also affect other organs (such as the liver and brain) and their metabolism and molecular responses, which, in turn, may interfere with colitis-underlying mechanisms and drug response, and may influence the interpretation of results. These intrinsic (intra-experimental) characteristics of the DSS model are summarised in the paper (colitis, gut–brain axis, gut–liver axis). In addition, the DSS model is heavily influenced by numerous extrinsic (inter-experimental) factors (environmental, microbiological, genetic), which may further complicate the colitis model, the study outcomes, and data interpretation, and these are also discussed in the paper. As science advances and new data accumulate, understanding the intricate interplay among internal mechanisms, external factors, and technical variables becomes increasingly essential for the accurate interpretation of DSS outcomes. This review synthesises the complexity and interdependence of factors shaping the DSS model, emphasising the need for meticulous reporting and consideration of methodological nuances to enhance reproducibility, interpretation, and translational value in DSS colitis research. In addition, the review provides practical guidance through a “traps and tricks” subsection and checklist table designed to provide a framework and practical recommendations to better understand, apply, and interpret DSS model results in the context of broader systemic and methodological considerations.

## 1. Introduction

Inflammatory bowel disease (IBD) is a group of chronic, immune-mediated multifactorial disorders characterised by relapsing inflammation of the gastrointestinal tract. The two main forms are Crohn’s disease (CD), which can affect any part of the digestive tract, and ulcerative colitis (UC), which is limited to the colonic mucosa. IBD is a lifelong disease occurring early in life in both men and women. Due to its early onset, chronic nature, and relatively low mortality rates, and due to the ageing population and increasing global incidence of IBD (10.5 to 46.14 newly diagnosed per 100,000 inhabitants every year in the EU, with CD ranging from 4.1 to 22.78 and UC from 3.0 to 23.36 per 100,000), the prevalence of IBD is rising significantly worldwide (187 to 832 per 100,000 in the EU, 61.6 to 178 per 100,000 for CD, and 99.84 to 191.4 per 100,000 for UC), contributing to an increasing burden on the global health care system [1,2,3]. In addition, IBD is often associated with prolonged abdominal pain and extraintestinal manifestations, indicating the complex nature of the disease. Patients with IBD present several clinically challenging problems for physicians. Despite recent treatment advancements, an efficient, safe and well-tolerated therapy to maintain long-term remission and pain relief is needed [4,5,6,7].

Despite decades of intensive research, the exact aetiology remains unknown. However, the interplay among genetic predisposition, environmental factors, microbiota, and immune response is importantly involved in the pathogenesis of IBD [8,9].

Due to the complexity of the disease, numerous animal models of IBD have been developed, including spontaneous colitis models, adoptive transfer models, genetically modified models, and inducible colitis models [10,11]. Among them, the dextran sodium sulfate (DSS) colitis model is the most widely used, with over 7000 PubMed entries in the last decade and an exponential increase in recent years. Its wide use is due to its simplicity (administration in drinking water) and versatility. The onset, duration, and severity of inflammation can be easily managed by adjusting DSS concentration and duration and controlled by monitoring clinical signs. An acute, chronic, or relapsing model can be produced by varying the concentration, duration, and frequency of DSS administration. Due to its simplicity, the DSS colitis model is also frequently induced in combination with other disease models, such as colorectal cancer (dimethylhydrazine (DMH)/azoxymethane (AOM)) [12,13,14] or non-alcoholic fatty liver disease (NAFLD) [15,16], to explore molecular mechanisms underlying the comorbidity of IBD with other diseases.

However, the multifactorial nature of the DSS colitis model (as in IBD) is both an advantage and a “curse” for researchers, as numerous factors significantly affect the model, the quality of a study, the interpretation of results, and translatability [17,18]. This is further complicated by the lack of information in publications [19].

**Figure 1:**
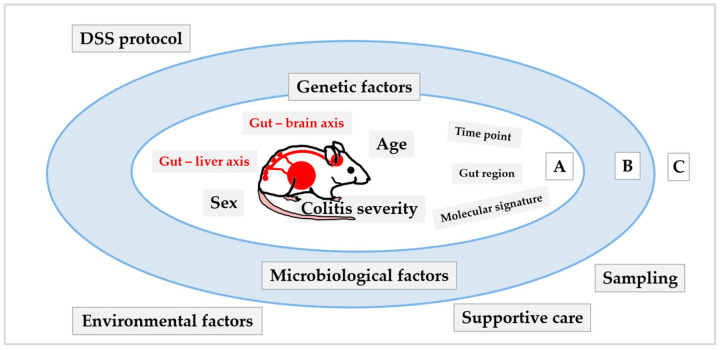
Schematic presentation of the main groups of intrinsic (intra-experimental; A) and extrinsic (inter-experimental; B, C) factors of the DSS model presented in the review.

Therefore, the present paper aims to illustrate the complexity of the DSS model, given its multifaceted nature, to better understand the interplay, interconnectedness, and interdependence among internal mechanisms and external factors (including technical traps) that affect the quality of the study, particularly in light of interpretation and reporting results, to highlight the importance of the details needed to evaluate study outcomes in the DSS model. To our knowledge, this is the first review to synthesise insights into the mechanisms triggered in the liver and brain following colitis induction together with the complexity of the DSS model (intra- and inter-experimental factors; Figure 1). In addition, the review provides practical guidance through a “traps and tricks” subsection after each section. At the end, information culminates in a consolidated checklist table designed to provide a framework and practical recommendations to better understand, apply, and interpret DSS model results in the context of broader systemic and methodological considerations.

## 2. Intrinsic Characteristics of the DSS Model (Intra-Experimental)

DSS colitis was first reported in 1985. Since then, it has been systematically evaluated in various species, with mice and rats being the most frequently used [20,21].

The broad use of the DSS-induced colitis model yields numerous insights into the model, helping better understand its complexity, characteristics and limitations. Although DSS colitis is induced locally, inflammation in the colon and gut barrier destruction may also affect other organs (such as the liver and brain) and their metabolism and molecular responses, which may, in turn, interfere with colitis-underlying mechanisms and drug response, and may influence the interpretation of results. These intrinsic (intra-experimental) characteristics of the DSS model are summarised in the first part of the paper (colitis, gut–brain axis, gut–liver axis). In addition, the DSS model is heavily influenced by numerous extrinsic (inter-experimental) factors (environmental, microbiological, genetic, etc., discussed in the second part of the paper), which may further complicate the colitis model, the study outcomes, and data interpretation.

### 2.1. DSS Colitis and Gut (Inflammation)

**Figure 2:**
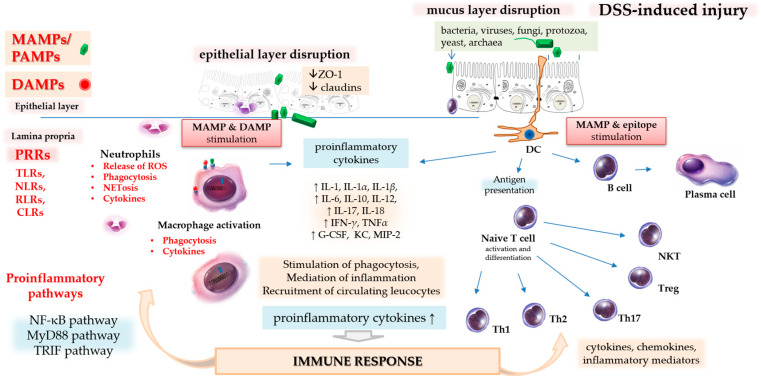
DSS-induced injury in the gut mucosal barrier. Disruptions in gut epithelial barrier integrity (mucus and epithelial layers) allow microbiota and luminal antigens to reach epithelial and immune cells, triggering innate and adaptive immune responses. Enterocytes and immune cells are equipped with different types of PRRs (i.e., TLRs, RLRs, NLRs, ALRs, CLRs), which are activated by MAMPs/PAMPs and DAMPs and trigger signals that recruit phagocytes and lymphocytes into injured tissue to combat invading microbes and injured cells. Antigen-presenting cells (DC) become activated upon recognising microbial epitopes and trigger the activation and differentiation of naïve T cells into specific CD4^+^ T cells or CD8^+^ cytotoxic T cells, and B cells into plasma cells. Legend: CLRs—C-type lectin receptors; DAMPs—damage-associated molecular patterns; DC—dendritic cell; MAMPs—microbial-associated molecular patterns; MyD88—myeloid differentiation primary response 88; NET—neutrophil extracellular trap; NF-κB—nuclear factor-44 κB; NOD—nucleotide-binding oligomerization domain; NLRs—NOD-like receptors; NKT—natural killer T cells; PAMPs—pathogen-associated molecular patterns; PRRs—pattern recognition receptors; RLRs—retinoic acid-inducible gene-I-like receptors; ROS—reactive oxygen species; Th1—T helper cells type 1; T-reg—regulatory T cells; TLRs—Toll-like receptors; TRIF—TIR-domain-containing adapter-inducing interferon-β.

DSS disrupts the integrity of the gut epithelial barrier (mucus and epithelial layers), thereby increasing mucosal permeability and allowing microbiota and luminal antigens to reach epithelial and immune cells (Figure 2) [21].

#### 2.1.1. Epithelial Barrier and Protective Mechanisms in the Healthy Colon

The mucus barrier in the colon consists of an outer, loose layer formed of gel-forming mucins secreted mainly by goblet cells and an inner, dense layer attached to the epithelial surface produced by enterocytes (glycocalyx). Gut microbes can be found in the outer mucus layer, while the inner layer is sterile [22]. Enterocytes and immune cells express different types of pattern recognition receptors (PRRs; i.e., Toll-like receptors (TLRs), retinoic acid-inducible gene-I-like receptors (RLRs), nucleotide-binding oligomerization domain (NOD)-like receptors (NLRs), and C-type lectin receptors (CLRs)). PRRs recognise microbe-specific components known as microbial-associated molecular patterns (MAMPs or PAMPs; pathogen-associated molecular patterns) and self-derived molecules generated from damaged cells, damage/danger-associated molecular patterns (DAMPs). Among the most studied PRRs are TLRs, transmembrane proteins expressed on the cell surface and in intracellular compartments of various epithelial and immune cells [22,23,24]. For instance, TLRs 1, 2, 6 and 10 are expressed on enterocytes, macrophages, dendritic cells, natural killer cells, mast cells, T cells, B cells and neutrophils, TLR4 is expressed on macrophages, dendritic cells, mast cells, natural killer cells and enterocytes, and TLR5 is expressed on Paneth cells, enterocytes, dendritic cells and macrophages [23,25]. TLRs are differently expressed along the length of the intestine (gut regions, cellular compartment and cell type-specific TLRs distribution) [26]. TLRs interact with their respective MAMPs/PAMPs and DAMPs. TLRs 1, 2, 4, 5, and 6 bind to components of microbial cell walls and membranes, such as lipopolysaccharide and lipoteichoic acid from cell walls, lipoproteins from the cell membrane, and a structural component of bacterial flagella called flagellin. TLRs 3, 7, 8, and 9 bind to microbial nucleic acids, including double and single-stranded RNA from RNA viruses and DNA from most organisms. However, TLRs cannot distinguish between foreign and self-nucleic acids (host cell nucleic acids). Recognition of foreign nucleic acids mainly depends on the location in the cell [25]. Other PRRs are NOD-like receptors or NLRs, located in the cytoplasm of cells for the detection and elimination of intracellular invaders [27]; cytosolic RLHs, which detect viruses [28]; transmembrane and soluble forms of CLRs, which detect fungi [29]; and secreted receptors such as ficolins [30] and pentaxins [31], which activate innate defence mechanisms involving complement and phagocytosis.

When gut microbiota (bacteria, viruses, fungi, protozoa) or luminal antigens come into contact with enterocytes and immune cells in the gut, this complex protective mechanism is activated, triggering innate and adaptive immune responses to eliminate intruders and repair damage (Figure 2).

#### 2.1.2. DSS Disrupts the Epithelial Barrier and Triggers an Immune Response

**Figure 3:**
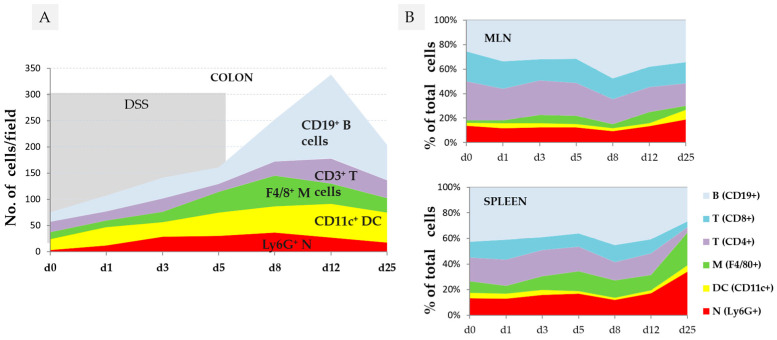
Schematic presentation of a mucosal (colon) and systemic (spleen and mesenteric lymph nodes-MLN) immune cell response (activation and recruitment of neutrophils (N), macrophages (M), dendritic cells (DC), and T and B cells) in DSS colitis in a temporal fashion. Innate and adaptive immune responses are induced during DSS colitis. (**A**) A progressive influx of phagocytes (neutrophils and macrophages) into the colon begins on day 1, peaking on day 8 (active colitis). After that, phagocyte numbers gradually decline, while adaptive immune cells increase, reaching a peak around day 12, and then gradual decrease as colitis progresses to a chronic stage (day 25), when significant numbers of T cells, B cells, and dendritic cells remain active. (**B**) In MLN, early immune activation is evident from day 1, affecting all major immune populations. DCs exhibit increased activation, B cells increase in both frequency and activation status, while T cell percentages decrease, although their activation (CD69 expression) is enhanced. During the chronic phase, there is an increase in innate immune cells (neutrophils and macrophages), accompanied by sustained production of proinflammatory cytokines. In the spleen, a transient increase in T cells is detected as early immune alterations. As the disease progresses, there is a shift toward innate immunity, with a significant decrease in T and B cells and a concomitant increase in neutrophils and macrophages during the chronic phase. (DSS protocol: C57BL/6OlaHsd females, 3% DSS (45 kDa) for 6 days) (adapted from [35]).

The first changes in the colon can be observed within 12 h of DSS consumption, when bacteria reach the epithelial cells [32]. One day after DSS consumption, alterations in tight junction proteins (zonula occludens-1 (ZO-1) [33], the production of proinflammatory cytokines (*Tnf-α*, *Il-1β*, *Ifn-γ*, *Il-10*, *Il-12*) [34], and the recruitment of neutrophils, dendritic cells, macrophages, and T and B cells in the colon wall, mesenteric lymph nodes and spleen were observed (Figure 3) [35].

Epithelial and immune cells in the gut, equipped with different types of PRRs (sensors for microbes), recognise and interact with their respective MAMPs/PAMPs and DAMPs, which trigger innate immune responses [22,36]. In addition, contact with microbes also stimulates the adaptive immune system through epitopes (different parts of microbial molecules with adaptive immune-stimulatory potential) presented by antigen-presenting cells, such as dendritic cells, to T and B cells. Released cytokines in turn trigger the differentiation of T and B cells and the production of proinflammatory cytokines to activate the inflammatory process further and recruit circulating leucocytes (first, mainly phagocytes (neutrophils and macrophages); later, mostly lymphocytes (T cells, B cells, and dendritic cells)) [35] to combat the microbes and repair the injury (Figure 2).

The activation of innate immunity in DSS colitis involves TLRs. The deletion of TLR signalling genes worsens DSS colitis in mice. TLR2, TLR4, and Myd88 promote epithelial proliferation and barrier restoration, while deleting TLR4 and Myd88 reduces neutrophil infiltration by lowering key neutrophil-recruiting chemokines [37]. TLR2 ligands or epithelial TLR4 activation may reduce the severity of DSS-induced colitis by upregulating interleukin (IL)-10 or granulocyte–macrophage colony-stimulating factor (GM-CSF), respectively, highlighting factors in the recovery phase of DSS-induced colitis [38,39]. Epithelial damage and microbial products activate the NOD-like receptor family, pyrin domain containing 3 (NLRP3) and other inflammasome components (via TLR activation), leading to the caspase-1-dependent processing of IL-1β and IL-18 [40].

A variety of histological changes can be seen in the course of DSS colitis development, such as mucin depletion, epithelial degeneration, a vacuolar hydropic degeneration of epithelial cells and infiltration of neutrophils into the lamina propria, cryptitis (transepithelial migration of neutrophils into mucosal epithelium), and crypt abscesses (migration of neutrophils through mucosal epithelium into crypt lumen and the formation of crypt abscess), leading to disappearance of epithelial cells. Histologically, DSS colitis can range from mild inflammation limited to the mucosa or submucosa to erosions or severe inflammation that penetrates all layers (transmural) of the colon wall [17]. On the molecular level, acute colitis is a highly complex and dynamic process that reflects functional and morphological perturbations in the colon sample in both a temporal and spatial manner [34,41,42,43]. Numerous inflammatory mediators have been implicated in the pathogenesis of human IBD and DSS colitis, including reactive oxygen species (ROS), cytokines, eicosanoids, nitric oxide, and activation products of the complement system and inducible nitric oxide synthase. More detailed molecular mechanisms of DSS colitis can be found elsewhere [11,17,21].

#### 2.1.3. Factors Affecting Molecular Signatures of DSS Colitis

**Figure 4:**
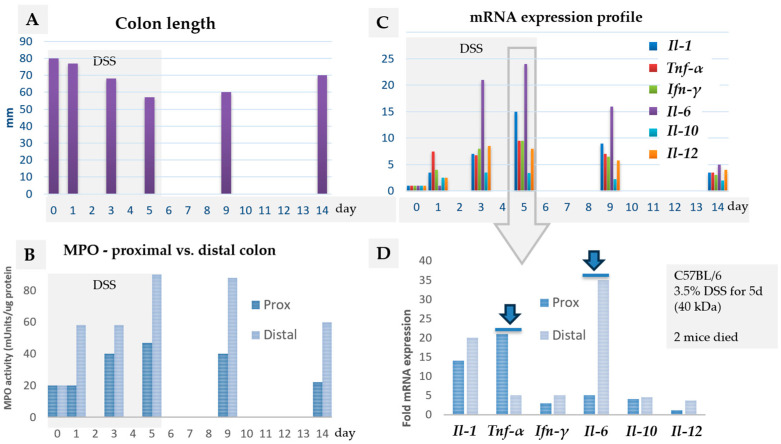
Temporal and spatial changes in clinical and molecular parameters during DSS colitis (DSS protocol: C57BL/6 mice; 3.5% DSS (40 kDa) for 5 days) (adapted from [34]). (**A**) Colon length during colitis induction and recovery. (**B**) Myeloperoxidase (MPO) activity in the proximal and distal parts of the colon. Higher activity of MPO in the distal colon indicates increased injury. (**C**) Proinflammatory mediators were determined in the whole colon length during colitis development and recovery. However, on the 5th day (a grey arrow), a separate analysis of the proximal and distal parts was also done. (**D**) Systematic analysis of the expression of proinflammatory mediators revealed distinct profiles in the proximal and distal parts of the colon (blue arrows show significant difference), highlighting the importance of a pre-planned sampling design.

**Figure 5:**
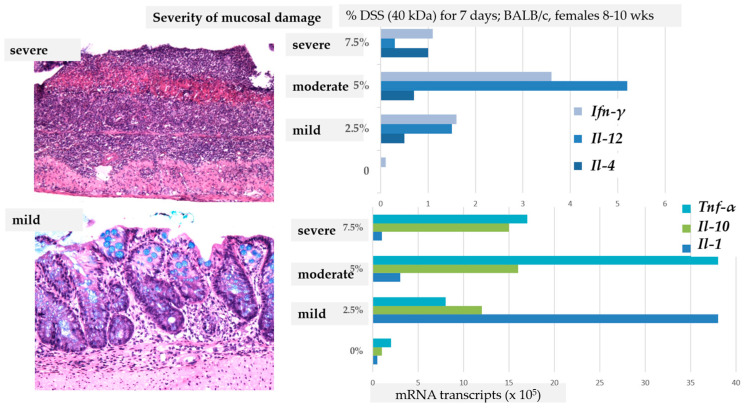
DSS protocol (concentration; 2.5%, 5%, 7.5%) affects the severity of injury and mucosal damage. Clinical and histological manifestations correlate with colitis severity (the higher the severity, the worse the clinical and histological scores) in the acute phase. However, molecular signature (i.e., chemokine profiles *Il-1*, *Il-4*, *Il-10*, *Il-12*, *Ifn-γ*, *Tnf-α*) in severe colitis differs (adapted from [48]).

Various intrinsic factors can affect the cellular and molecular signature of DSS colitis, such as the phase of the colitis (acute, subacute, chronic) [17,44,45] and the age of animals [46,47]. The expression of molecular factors depends on the time point of sampling or measurements during the course of colitis (Figure 4C) [34,42], the location or gut region of sampling (Figure 4B,D) [34], the molecular factor analysed (Figure 4C), and the severity of DSS colitis (Figure 5) [48], including the reference genes used [18,49].

#### 2.1.4. The Severity of Colitis

DSS colitis severity and progression are traditionally assessed using the Disease Activity Index (DAI) (i.e., scoring body weight loss, faecal consistency, and the presence of faecal blood) [50]. In general, it is believed that the production of inflammatory mediators increases progressively during the acute phase of DSS colitis, correlating with clinical manifestations. However, while an increase in histological scoring correlates with clinical manifestations (DAI), the expression of molecular factors does not necessarily correlate with increases in the histological score. For instance, the progressive upregulation of chemokines (*Il-12*, *Ifn-γ*, *Il-1*, *Tnf-α*) correlated with the degree of inflammation in mild or moderate colitis, but not in severe colitis, where chemokine expression was significantly lower (Figure 5) [48]. We have shown that the expression of some molecular factors may differ in regard to the histological features of DSS colitis (i.e., mild mucosal inflammation vs. erosion) [18]. Histologically, in mild colitis, the increased infiltration of inflammatory cells in the mucosa and mucin depletion are evident; epithelial cells are injured and diminished, but still present. In severe colitis, the epithelial layer is damaged, leading to the total disappearance of epithelial cells (erosion) and massive infiltration of phagocytic cells, mostly neutrophils, resulting in a distinct molecular signature [51].

#### 2.1.5. Reference Genes in DSS Colitis: mRNA/miRNA

The selection of reference genes is another important factor that can contribute to contradictory results and variability among studies.

In DSS colitis, the stability of mRNA reference genes was evaluated on fresh colons of C57BL/6J males [49] and formalin-fixed and paraffin-embedded (FFPE) colons of C57BL/6JOlaHsd mice (males and females) [18]. Among 13 candidate mRNA reference genes evaluated, a combination of *Eef2*/*Tbp* was selected as an mRNA reference gene for fresh colon samples [49], and a combination of *Eef2*/*Rplp0* for FFPE colon samples of C57BL/6 mice [18]. As reference genes for miRNA, a combination of miR-191-5p/miR-16-5p was selected instead of the highly unstable U6 [18].

It has been shown that the stability of reference genes, like *Actb*, *Β2m*, *Hmbs*, *Hprt*, and *Gapdh*, is significantly affected by DSS colitis, and their use in DSS studies is strongly discouraged because it can lead to misleading and conflicting results [49].

**Figure 6:**
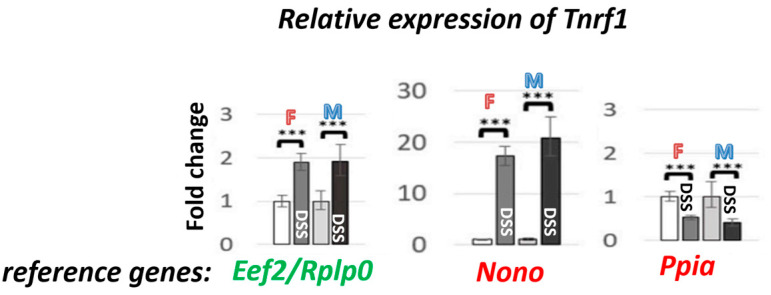
Example of how the reference gene affects the expression level (adapted from [18]). Tumour necrosis factor receptor 1 (*Tnfr1*) was overexpressed in DSS colitis when *Nono* or a combination of *Eef2*/*Rplp0* was used as a reference gene. However, there was a 10-fold difference in *Tnfr1* expression levels. When *Ppia* was used as a reference gene, *Tnfr1* was found to be downregulated in DSS colitis. *Nono* and *Ppia* (red color) have been found as unstable reference genes, while a combination of *Eef2*/*Rplp0* (green color) has been found as the most stable for colon samples in DSS colitis. Legend: F—females; M—males; *** *p* ≤ 0.001.

The graph (Figure 6) shows real data on how the selection of reference genes can affect *Tnfr1* expression levels, using the most stable (a combination of *Eef2*/*Rplp0*) and least stable (*Ppia*, *Nono*) reference genes in DSS colitis. The results vary significantly, not only in the expression levels (upregulated, with a 10-fold difference between *Eef2*/*Rplp0* and *Nono*) but also in the direction of regulation (downregulation in the case of *Ppia*).

However, other intrinsic factors affecting cellular and molecular signatures, such as histological features, the microenvironment, and sampling methods, were not studied. With advances in technology, these factors can also be assessed and deciphered in the near future. FFPE samples from animal studies represent great potential for research, particularly because pathology is an essential part of the study, and the histological picture has been shown as a significant factor influencing the absolute expression levels of target genes [18]. FFPE samples are an invaluable source for RNA research (e.g., retrospective analysis, biomarker identification, and evaluation of robust reference genes) without the need for additional animal use.

#### 2.1.6. Traps and Tricks

In severe DSS colitis, injury is typically diffuse, resulting in severe inflammation and erosion that involves the entire colon. In contrast, in mild colitis, inflammation is typically focal, localised to a smaller region of the colon, with intact areas between the damaged sections. Therefore, longitudinal sections are more representative of the severity of DSS colitis than cross-sections. However, when a colon is used for various analyses, sampling and tissue preparation for further analyses may be among the reasons for discrepancies within and among studies due to all the above-mentioned factors. Therefore, it is recommended to keep sampling methods consistent throughout the study to minimise the variability of cellular and molecular factors and always collect the same part of the colon for each analysis. In addition, to minimise circadian effects on molecular parameters, it is recommended to perform all procedures, sampling, and euthanasia at the same time of the day within a narrow time window across all groups throughout the study [52].

### 2.2. DSS Colitis and Abdominal Pain (Gut–Brain Axis)

Over 70% of people with IBD suffer abdominal pain during active flares of colitis. The pain can persist even when the disease is in complete endoscopic remission (post-inflammatory abdominal pain or visceral hypersensitivity) [4,53,54]. Severe abdominal pain and hypersensitivity worsen emotional well-being, cognitive function, and mood, reducing quality of life [4,55,56]. Current treatments for IBD-related pain and related psychiatric symptoms are often ineffective or have harmful side effects. Thus, the treatment of abdominal pain and hypersensitivity in IBD remains an important clinical challenge [57]. The mechanism responsible for abdominal pain, hypersensitivity and pain-related depression behaviour is under intense investigation [58,59,60]. Recent studies have demonstrated complex multiple pathways between the gut and the brain [5,61], linking IBD (dysbiosis, alteration in immune signals, damaged tissue, inflammatory cells, etc.) with cognitive impairment and depression [55,56].

Abdominal pain is a complex process that involves the enteric nervous system (ENS; intrinsic and extrinsic), the central nervous system (CNS), the gut immune system, the epithelial barrier system, and the gut microbiota [5,62]. The intrinsic ENS (neurons that reside within the myenteric and submucosal plexus) is involved in gut motility, secretion, and immune and endocrine functions. Nociceptors, a specialised subset of sensory neurons of the intrinsic ENS, are equipped with numerous receptors to detect various stimuli in the gut, including inflammatory mediators (histamine, proteases, serotonin), chemokines (IL-1β, IL-6, IL-10, TNF-α), and neuropeptides (substance P; SP, calcitonin gene-related peptide; CGRP) that are released from damaged gut cells, immune cells or microbiota. These molecules bind to receptors and activate channels (G protein-coupled receptor; GPCR, transient receptor potential channels; TRPs) on the peripheral endings of enteric neurons, which, in turn, release neuropeptides (CGRP, SP, and others) into the colonic mucosa to maintain immune and microbial homeostasis. At the same time, nociceptors, in response to these stimuli, depolarise nerve endings, activate gut afferent neurons, and transmit pain signals to the central nervous system, where the pain is perceived. When nociceptors are sensitised, the previously ineffective stimuli become effective and result in aberrant pain perception (central sensitization). Immune cells, epithelial cells and the gut microbiota all communicate with sensory neurons and interact and modulate the gut–brain axis [5,62]. The gut microbiota influences the brain by producing metabolites (e.g., short-chain fatty acids, tryptophan metabolites) and neurotransmitters (e.g., gamma-aminobutyric acid; GABA, serotonin) and modulating immune and inflammatory responses [63,64]. In addition, enterochromaffin cells (excitatory enteroendocrine and neuroendocrine cells, which produce more than 90% of the body’s serotonin, although they represent less than 1% of total intestinal epithelia) can also trigger or exacerbate pain, even without inflammation, in response to various stimuli (nutritional, microbial metabolites, mechanical stretch, stress-induced hormones, neurotransmitters). Enterochromaffin cells have a long basolateral projection (termed a neuropod) and can form synaptic-like connections with nerve fibres, making them a direct line of communication between the gut epithelium and specific primary afferent nerve fibres (involved in physiologic responses such as emesis, motility, and visceral pain) [65,66,67]. This complex communication can lead to peripheral sensitization and the modulation of visceral pain processing (dysregulation of brain–gut interaction).

#### 2.2.1. Abdominal Pain in DSS Colitis

It has been shown that acute DSS colitis induces several pain-related molecular changes in the colon (Trpv1, SP, Cgrp, Iba-1) [68,69,70,71] and dorsal root ganglia of the spinal cord (Trpa1, pp42/44 Mapk, cFos) [72,73,74] and in brain regions such as the thalamus, hypothalamus, amygdala, and prefrontal cortex (cFos, a marker of neural activation) [73].

Recent studies have identified novel targets implicated in DSS colitis abdominal pain, such as transient receptor potential melastatin 3 (Trpm3), a subgroup of TRP channels found to be involved in inflammatory bladder and joint pain [74], or sigma-1 receptors (σ_1_R) that have been identified in several pain pathways [75].

Acute DSS colitis elicited mechanical hypersensitivity in the abdominal skin (termed referred hyperalgesia due to the overlap of visceral and somatic nerves in the spinal cord) and in the plantar and facial skin, suggesting central sensitization and widespread sensitivity [72]. Interestingly, in mice with a histologically recovered colon (35 days after DSS), some pain-related markers remained elevated in the colon (Trpv1, SP) and dorsal root ganglia (Trpv1) [70], and mechanical hypersensitivity of the abdominal skin was still present, suggesting post-inflammatory hypersensitivity (persistent intestinal hyperalgesia) [68,69] (Table 1).

Recent studies have shown that the myenteric plexus, an important structure for the transduction of pain signalling in the colon wall, can also be injured in DSS colitis. Acute DSS colitis leads to blood–myenteric barrier disruption and increased numbers of intraganglionic macrophages, which allows inflammatory stimuli to access the myenteric plexus and cause neuroinflammation [76]. Myenteric barrier disruption and subsequent enteric neuronal injury, associated with neuroinflammation and neurodegeneration (overexpressed Bax1, Hdac4, Il-18, Casp8, Hif1a), can lead to gut dysmotility [77]. In the chronic DSS colitis model, an increased infiltration of monocytes, B cells and CD8+ T cells was observed in the myenteric plexus (as in IBD patients) [78]. Interestingly, an alteration in the morphology and expression profile of the perineuronal net-like structure around dorsal root ganglia neuronal cell bodies was also found in DSS colitis [79].

**Figure 7:**
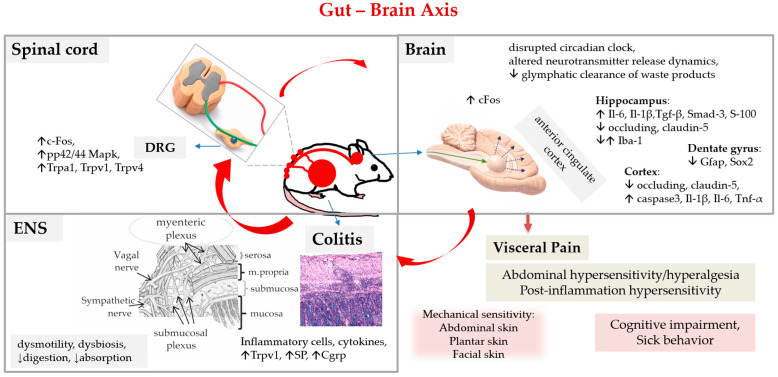
Alterations observed in the ENS and CNS of animals with acute DSS colitis (myenteric plexus, DRG, microglia, different brain regions involved in modulation and pain perception). Pain is driven by the activation of visceral nociceptors in response to gut inflammation; these depolarise the nerve terminals and transmit pain information to the CNS. CNS prolongs or amplifies the sensitisation of visceral afferents (red arrows), contributing to chronic abdominal pain (central sensitisation and visceral hypersensitivity) and sick behaviour. ↑—denotes increase; ↓—denotes decrease.

Evidence shows that peripheral inflammation (DSS colitis) and immune signalling can cause morphological or functional disruption of the blood–brain barrier (i.e., changes in tight junction, damage to endothelial cells, activation of glia cells, penetration of peripheral immune cells, alteration of transport pathways and receptors) and affect brain function and mood [80,81,82]. Interestingly, mice with acute DSS colitis and increased cytokines in plasma (Il6, Il-18, Tnf-α, Gro-α) showed alterations in blood–brain barrier tight junction proteins (occludin and claudin-5) [83], elevated inflammatory-related molecules in the brain (*Gro-α*, *Tnf-α*, *Il*-*6*, *Il*-*1β*, *S*-*100*, *Tgf*-*β* and *Smad*-*3*) [71,83,84] and activation of glia cells (Iba-1, Gfap, Sox2) [71], suggesting neuroinflammation and a disruption of the blood–brain barrier. It was also demonstrated that acute DSS colitis can induce elevated levels of corticosterone in blood and alterations in neuropeptide expression in distinct brain regions (*Npy*, *Npy1r*, *Crh*, *Crhr1*, *Bdnf*, *Nr3c1*) (Figure 7) [84].

Recent evidence shows that microglia, the resident immune cells of the CNS (brain and spinal cord), are importantly involved in DSS colitis visceral hypersensitivity. In the spinal dorsal horn, microglia were found to be activated by the macrophage colony-stimulating factor (M-CSF) produced by spinal astrocytes in DSS colitis (D28). Activated microglia (c-Fos, NeuN), in turn, released inflammatory factors in the spinal dorsal horn (Il-6, Il-1β, Tnf-α) [85]. In the anterior cingulate cortex, a region involved in pain modulation and related emotional processing (such as anxiety and depression), microglial innate immune receptors Trem-1 and Trem-2 (triggering receptors expressed on myeloid cells-1/2) were found to be involved in modulating visceral hypersensitivity [86].

A recent study reported that mice with acute DSS colitis have a disrupted circadian clock and altered CNS fluid distribution, reduced glymphatic clearance of waste products and altered neurotransmitter release dynamics [87].

Interestingly, it was also found that chronic DSS colitis alters taste input to the brain (reduced neural taste responses to natural and artificial sweeteners due to modulation of the expression of receptor subunits that transduce sweet and umami stimuli in oral taste buds) [88], which is information to pay attention to in behavioural studies involving taste. The number of studies using DSS colitis as a model of visceral pain is increasing, showing DSS colitis as an attractive model for studying the underlying mechanisms of visceral pain. The DSS colitis rat model was already evaluated for its ability to mimic chronic intestinal hypersensitivity and has been recognised as appropriate for use in the characterisation of new pharmacological treatments against visceral pain [89].

#### 2.2.2. Gut-Brain Axis as a Potential Confounding Factor

The gut–brain axis is a bidirectional communication system linking the gastrointestinal tract and the CNS through neural, hormonal, and immune pathways. Signals generated in the gut (nerve signals via the enteric nervous system and vagus nerve, gut hormones, inflammatory molecules, and microbial metabolites) can influence brain function, mood, stress responses, and cognition. However, the CNS can modulate gut physiology (gut motility, secretion, and even the gut’s immune activity) via stress responses (Hypothalamic–Pituitary–Adrenal axis) and autonomic nervous system outputs. It has been shown that psychological stress may modulate visceral pain [84] and may have a confounding effect on DSS colitis, from no effect [73] to a significant effect [90,91].

Since gut–brain communication is mediated by multiple pathways (neural, hormonal, and immune), it can also operate on different time scales (seconds to minutes for neural signals, minutes to hours for hormones, days for immune signals), potentially introducing an additional dimension into the DSS colitis model. However, these pathways remain poorly characterised in the DSS colitis model, and further studies are required to elucidate their roles, interactions, and temporal dynamics.

In addition, it is important to keep in mind that various molecular factors may play dual roles in inflammation. For instance, transient receptor potential ankyrin 1—TRPA1, a receptor involved in inflammation and pain and a subject of debate in the past—has been found to have a dual role in colonic inflammation: a proinflammatory role in the acute phase (primarily via innate immune cells) and an anti-inflammatory role in the subacute phase (by modulating adaptive immunity) [92].

#### 2.2.3. Spontaneous Behaviour in DSS Colitis

Spontaneous behaviour that accompanies visceral pain/discomfort in DSS-treated animals was assessed in some studies. One study reported no changes in the daily activities of mice with acute mild colitis compared to healthy controls [93], another reported reduced travel distance and less time spent climbing in the acute and post-inflammatory phase of colitis [70], and the third reported reduced locomotion and rearing during grooming (splash test) in acute colitis [84]. However, the methods used in the past differ significantly across studies; assessment was performed only at a single time point and without prior habituation of the animals, which very likely affected the results.

In recent years, more effort has been devoted to developing non-invasive assessment methods for spontaneous behaviour. Methods based on the assessment of animals’ appearance, such as the grimace scale (evaluating facial expression patterns) or composite behaviour (observing the presence of writhing, vertical back arching, stagger/fall, twitch, and belly pressing), have already been systematically evaluated in DSS colitis. The grimace scale, a reliable marker of pain in numerous models, has been evaluated as a sensitive marker of pain in the DSS colitis models in rats [94] but not in mice [95], while composite behaviour has not been found to be sensitive enough (although all behaviours except belly pressing were observed in acute DSS colitis) [94]. Methods that assess animals’ voluntary (positive) behaviour, such as burrowing, have also been shown to effectively identify pain in acute and chronic DSS colitis (in correlation with colitis severity assessed using the DAI) [94,96].

Novel, more refined methods that do not disturb animals (i.e., avoiding restraint, handling, or separation) and can detect more subtle signs (i.e., AI-supported methods that can allow the tracking of animals’ behaviour and their activity during the whole day, particularly during the night; nocturnal animals) are in progress and will be very helpful in the future after systematic evaluation. These include individual voluntary wheel running in group housing conditions (decline in positive behaviour) [97] or an automated home-cage monitoring system [98]. In the future, AI-based behavioural monitoring tools are expected to provide high-resolution, continuous datasets capturing complex and subtle behavioural patterns that are not detectable with traditional methods. Such approaches will enable a more objective and quantitative assessment of pain and disease progression.

#### 2.2.4. A Welfare-Based Intervention and Pain Medication in DSS Colitis

DSS colitis is associated with abdominal pain/discomfort. Less time spent climbing, borrowing, or rearing [70,71] shows that animals avoid stretching abdominal muscles. Tail handling or restraining animals for administration (medications) might thus be more unpleasant for DSS animals than healthy controls.

As a welfare-based intervention to minimise animal suffering, various approaches were proposed, such as fluid supplementation [99] and pain medication [94]. However, fluid hydration (1 mL of 0.9% NaCl daily, ip injections) was found to affect the evolution of colitis by reducing clinical signs and accelerating epithelial repair, but not welfare, in DSS-treated mice. Thus, the routine use of fluid supplementation in DSS-treated mice is not supported [99]. The effect of paracetamol, tramadol, metamizole and buprenorphine on DSS-induced colitis in C57BL/6, CD1 and BALB/c mice was partially evaluated [100,101]. Fentanyl was found to exacerbate DSS colitis in C57BL/6J and BALB/c mice *via* Th1 cell- and macrophage-mediated mechanisms (i.e., increased μ-opioid receptor + Th1 cells and macrophages and increased Ifn-γ, Il-1a, G-csf, Rantes, Lix, Mip-1a, and Mip-1β), regardless of the dose [102]. Opioid hydromorphone led to barrier disruption, the translocation of bacteria, and increased intestinal and systemic inflammation, thereby aggravating DSS colitis in C57BL/6J mice [103].

The use of pain medication in DSS colitis is currently controversial due to the following reasons, which need to be taken into consideration:

- In CD patients, pain usually occurs 1–2 h after meals, while UC patients experience painful defecation, and the pain management is thus primarily regulated by the food intake [104].
- In IBD patients, clinical symptoms are treated through a suppression of the immune reaction (cortisone compounds, aminosalicylates, immunosuppressants, biologicals, Jak inhibitors), which does not include pain medication. If analgesics (NSAIDs, COX-2, cannabis) are used as pain relief, they are only for a short period of time, due to their controversial effects (longer use of analgesics can aggravate intestinal inflammation) [104].
- Analgesics have various effects on immune cells (i.e., granulocytes, macrophages and monocytes, lymphocytes) and impact underlying mechanisms, for example, binding to TLRs (opioids), dampening the activation state of T cells, regulating neutrophil adhesion and migration, and causing the aggravation of colitis in animals (excellently described in [104]).
- The use of analgesics has been shown to affect the gut microbiota, causing dysbiosis [105].
- Most of the analgesics are metabolised in the liver by drug-metabolising enzymes, whose activities are influenced by colitis in unpredictable ways (see next section).

#### 2.2.5. Traps and Tricks

Spontaneous behaviour involves peripheral and central sensitisation, as well as emotional factors (such as stress). Mice are prey animals and, as such, hide the signs of low or moderate pain when they are the subject of observation (even if a person is simply present in the room). If the animals must be moved from their home cage for behavioural testing, allow them time to habituate to the testing environment before entering the study. This helps separate actual pain from stress-related changes.

If pain relief drugs are used in DSS colitis as a welfare intervention, their effects need to be evaluated systematically before the main study:

- Test analgesics to find the appropriate drug, dose, and administration route for the specific strain and sex.
- Measure drug levels in blood to ensure the desired exposure is reached, as colitis can affect drug metabolism (explained in the next section).
- Test also potential side effects on colitis parameters, inflammatory mechanisms and microbiota (described in [104]).

### 2.3. DSS Colitis and Liver (Gut–Liver Axis)

IBD is associated with extraintestinal manifestations, such as hepatobiliary symptoms, that develop in up to 50% of patients with IBD [106], presumably due to gut barrier dysfunction (i.e., leaky gut) [107]. Studies have shown that, during acute DSS colitis, proinflammatory cytokines (Il-1β, Il-6, Tnf-α) can be elevated in the liver [108,109] without any change in liver histology or blood enzyme levels (ALT, AST). Given that at the same time (the active phase of colitis) bacterial lipopolysaccharide (LPS), a component of the outer surface of Gram-negative bacteria, was detected in the portal blood of animals with a disrupted mucosal barrier, it is assumed that the exposure of LPS (bacterial products derived from the intestine due to an increased gut permeability) triggered the activation of TLRs in Kupffer cells (liver macrophages), seen as the production and release of inflammatory cytokines [108,109]. Interestingly, the combined administration of a high-fat diet and DSS (C57BL/6) resulted in higher levels of LPS in portal blood, a higher gene expression of TLR4 and TLR9, and histologically confirmed mild inflammation in the liver [110]. Recent studies have also reported lipid metabolic disruption (i.e., fatty acid oxidation, lipogenesis, lipolysis) in the liver [15].

#### 2.3.1. Alterations in the Liver Affect the Pharmacokinetics of Drugs

DSS colitis has also been shown to affect hepatic metabolism [111] and the expression and activity of various drug-metabolising enzymes involved in the biotransformation of most drugs in clinical use. These include families of cytochrome P450 (CYP1, CYP2, CYP3) hepatic enzymes [108,109,112,113], factors involved in the regulation of these enzymes (NF-κB, pregnane X receptor—PXR, and constitutive androstane receptor—CAR) [108,112], and other phase I biotransformation enzymes, phase II biotransformation enzymes (UDP-glucuronosyltransferases; UGTs; UGT1A1 and 1A6), and drug transporters (Table 2) [113,114].

These changes in enzyme activity can modify the pharmacokinetics of certain drugs for IBD, impacting their therapeutic effectiveness or leading to drug-specific side effects, as observed in patients with UC (metronidazole [116] and cyclosporine [117]). Changes in hepatic metabolism and drug-metabolising enzymes have been shown to affect the pharmacokinetics and response of drugs in DSS colitis. For instance, higher plasma concentrations of tofacitinib, a drug for the chronic treatment of UC [112], or phenytoin, an antiepileptic drug [118], were found in DSS colitis male mice compared to healthy animals [112,118]. It was demonstrated that, in animals with DSS colitis, the pharmacokinetics of oral drugs differ for different drugs (lovastatin, simvastatin, pravastatin, cyclosporine D; drug specific impact) due to alterations in the activity of drug-metabolising enzymes [113].

#### 2.3.2. Traps and Tricks

It is important to take into consideration that the expression of drug-metabolising enzymes and variation in drug responsiveness can be significantly affected by various confounding factors, such as the phase of colitis (active/acute vs. chronic), the severity of colitis (mild vs. severe) [109], sexual differences [115,119,120], microbiological status (specific pathogen-free—SPF vs. germ-free—GF animals) and gut microbiome composition [121,122].

Therefore, when testing a drug dose or route of administration in the DSS colitis model, pharmacokinetic and pharmacodynamic studies should mimic the planned clinical use as closely as possible, including potential changes in drug-metabolising enzymes due to colitis.

If the test compound is administered orally in a separate drinking water bottle, it can alter thirst and fluid intake (DSS solution), which may affect colitis severity and impact the results.

## 3. DSS Colitis Variability and Reproducibility—Inter-Experimental Factors

**Figure 8:**
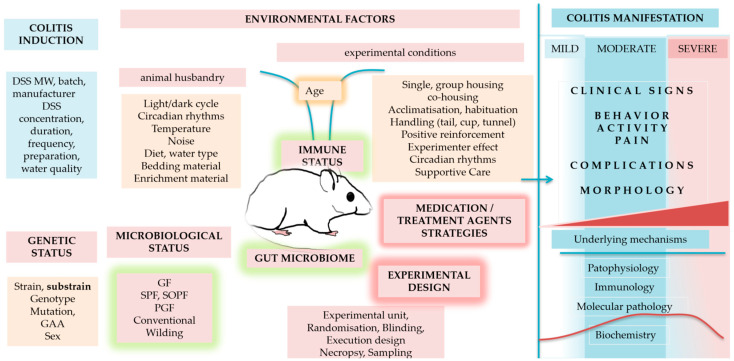
Schematic presentation of intra- and inter-experimental factors affecting DSS model, reproducibility, study outcome and interpretation of results. Intensive investigation of the molecular mechanisms of DSS colitis yielded a wealth of information, including contradictory findings. The latter is very likely the consequence of numerous confounding factors discussed in the present paper. Legend: GAA—genetically altered animals; GF—germ-free; SOPF—specific and opportunistic pathogen-free; SPF—specific pathogen-free; PGF—pseudo-germ-free.

Intra-experimental factors related to the induction and characteristics of the DSS model (DSS protocol (concentration, duration of DSS exposure), sampling, histology, colitis, pain, liver metabolism, enzymes, etc.) have been discussed in the previous sections. Inter-experimental factors that can significantly modulate the DSS model across studies and laboratories are presented in the next section. Intra- and inter-experimental factors are summarised in Figure 8.

### 3.1. Molecular Weight and Effectiveness of DSS

The molecular weight of DSS directly affects colitis induction, its severity (degree of inflammation), and the primary location of lesions (cecum, upper/proximal, middle, distal colon). DSS is a negatively charged polysaccharide with a highly variable molecular weight, ranging from 5 to 1400 kDalton (kDa). DSS, with a molecular weight of approximately 40 kDa (ranging from 36 kDa to 50 kDa), induces lesions in the colon [123], primarily localised in the middle and distal regions, with increased severity observed in the distal colon. DSS with lower molecular weights (5 kDa) tends to cause milder inflammation, primarily affecting the upper colon and cecum (5% DSS for 7 days; BALB/cCrSlc; singly housed), while DSS with a high molecular weight (500 kDa) fails to induce intestinal lesions or inflammation [123]. The molecular weight of DSS defines its 3D molecular structure and size, which influences its ability to pass through the intestinal mucus layer. DSS with a high molecular weight (↑ 100 kDa) cannot cross the mucus layer, while DSS with a lower molecular weight can more easily penetrate the mucosal layer [124].

The mechanism by which DSS passes through the mucosal epithelial cells (transcellularly or paracellularly, via tight junctions) is unclear. DSS can form complexes with microbe-derived medium-chain fatty acids (MCFAs), which are prevalent in the colonic lumen. Larger DSS molecules (40 kDa vs. 5 kDa) are more likely to form complexes with MCFAs. These DSS-MCFA complexes penetrate the mucus layer and fuse with colonocyte membranes. However, since the diameter of a DSS-MCFA complex is significantly larger (2 to 4 times) than that of the corresponding non-complexed DSS, larger DSS molecules (↑ 100 kDa) complexed with MCFA cannot penetrate the mucus layer [125].

In vitro, DSS can directly penetrate the intestinal mucus layer by reducing the thickness of the inner layer, thereby enabling bacteria to reach epithelial cells (observed within 15 min). In the colon of DSS-treated mice, bacteria reach the epithelial cells before any infiltration of inflammatory cells occurs (within 12 h after DSS consumption) [32]. In in vitro settings, DSS also increased the paracellular permeability of the colon epithelial layer. Transmission electron microscopy showed the formation of vacuole-like structures in the intercellular space between adjacent epithelial cells, suggesting an action of DSS on the tight junction between neighbouring colonocytes [126]. Within 1–2 h, DSS reached the lamina propria and accumulated in the cell nuclei of both the innate and adaptive immune systems (T cells, macrophages, mast cells, plasma cells, fibroblasts). The disruption of nucleosomes through interactions with histones was proposed to play a role in DSS colitis [126]. In the intestinal Caco-2 cell culture, DSS disrupts ER homeostasis by increasing the levels of ER stress protein markers (immunoglobulin-binding protein (BiP), C/EBP homologous protein (CHOP), activation transcription factor 4 (ATF4), and X-box binding protein (XBP1)). ER homeostasis disruption impairs intracellular protein and membrane trafficking, leading to changes in membrane integrity and cellular polarity, and consequently compromising epithelial barrier function [127].

DSS (40 kDa) that penetrates the intestinal mucosa is taken up by mononuclear phagocytes and is eliminated mainly through the urine. A day after DSS consumption, DSS was detected in macrophages within the colon wall, mesenteric lymph nodes (MLNs), and Kupffer cells (phagocytic cells in the liver sinusoids). Three days later, DSS was detected in macrophages in the spleen, and 7 days later it was detected in the epithelial cells of the proximal renal tubules in the kidney, Kupffer cells, and in mononuclear cells in the subcapsular sinus of the MLN. DSS was even found in the Kupffer cells 8 weeks after DSS cessation [128]. During the chronic phase of DSS colitis, considerable amounts of DSS were found in the spleen [128,129]. However, in the brain, lung, heart, thymus, stomach, and duodenum, DSS was not observed. The DSS that does not penetrate the intestinal mucosa is eliminated with faeces [129].

#### Traps and Tricks

DSS is resistant to degradation by intestinal microbiota, anaerobic incubation, and varying pH (4.0–7.5) [129]. However, a high decomposition of DSS was reported following autoclave treatment for sterilisation (70%) and under very alkaline conditions (30% of sulfate was depleted from DSS), but decomposition was low under acidic conditions (10%) [130]. The efficacy of the DSS solution can be significantly affected by the water quality and the DSS solution preparation. Tap water contains a diverse array of minerals and microorganisms, with fluctuations over time that significantly affect the effectiveness of DSS. Thus, use autoclaved or pre-filtered water in a storage tank so that all groups have the same water quality during the experiment, and prepare fresh DSS solutions daily (do not acidify the water (HCl) or autoclave DSS solutions, as acidic conditions or autoclaving decompose DSS). To avoid batch variability, it is recommended to purchase DSS in larger quantities and store it in a dry place (hydrophilic).

### 3.2. Genetic Factors in DSS Colitis

Historically, various mouse strains have been shown to exhibit significant variation in susceptibility to DSS treatment. There is a strain-specific response (the severity of inflammation and anatomical site of inflammation) [131] (Table 3), which reflects variations in the expression of molecular factors involved in these mechanisms and their interactions with the environment.

#### 3.2.1. Effect of Strain

For instance, CBA/CaJ mice are less susceptible to acute DSS colitis than the C57BL/6 mouse strain. The lower susceptibility in CBA/CaJ mice was associated with higher IgA levels and greater barrier protection against bacteria [132]. C3H mice are more susceptible to DSS colitis than CBA/H and BALB/c [133]. The C3H/HeJBir substrain, created through selective breeding for a spontaneous colitis phenotype, is highly susceptible to colitis due to the increased reactivity of B and T cells to gut microbiota antigens [134]. BALB/c mice exhibit differences in baseline levels of angiogenic factors and in their response to DSS colitis compared to C57BL/6 mice [135]. BALB/c mice secrete a distinct panel of cytokines in response to DSS colitis and immunological stimuli, compared to C57BL/6 mice [45].

#### 3.2.2. Effect of Genetic Background

The effect of the genetic background has been shown to play a crucial role in characterising the function of specific genes and their role in colitis (genotype–phenotype relationship). For instance, the deletion of the IL-10 gene resulted in severe colitis in 129/SvEv and BALB/c mice [136]. In C3H/HeJBir mice, the deletion of IL-10 led to severe cecal and colonic lesions that developed as early as 4 weeks of age, whereas IL-10 deletion in C57BL/6 mice resulted in mild colitis with a delayed onset [137,138].

#### 3.2.3. Effect of Substrain

Even subtle genetic differences, such as those between C57BL/6 substrains (Table 4), can significantly impact study outcomes and the interpretation of results. For instance, when NOD2^−/−^ mice with the C57BL6NHsd genetic background were developed, numerous alterations to the B cell compartment (i.e., multiple B cell defects, deficiencies in recirculating B cells, marginal zone B cells, B1a cells, etc.) were observed and attributed to *Nod2* deletion and inflammatory bowel disease susceptibility. However, subsequent studies reported the absence of such an alteration in other NOD2^−/−^ mice. Finally, it was discovered that C57BL6NHsd mice carry a *Dock2* mutation, which was responsible for the observed effects in NOD2^−/−^ mice [139]. Since the *Dock2* mutation affects B cells (and B cells are involved in IBD and DSS colitis), the use of the C57BL6NHsd substrain might affect DSS colitis. The *Crb1^rd8^* mutation in the C57BL/6N mouse has implications for vision research [140] and may affect behavioural tests involving vision.

Mutations in the *Snca* gene in C57BL/6JOlaHsd (the absence of α-synuclein, known to be involved in Parkinson’s disease) were demonstrated to contribute to variations in behavioural (anxiety-like) and neurochemical differences and even the responses of glial integrity in substantia nigra and caudate putamen to treatment (Table 5) [141]. It was reported that chronic mild gut inflammation accelerates brain neuropathology and motor dysfunction in genetically engineered α-synuclein mutant mice [142]. Thus, the use of the C57BL/6JOlaHsd substrain (which harbours spontaneous Snca and Mmrn1 mutations) in DSS colitis may affect behavioural tests, abdominal pain studies, or even colitis outcome.

#### 3.2.4. Effect of Sex

Evidence shows that sex, often neglected in DSS model studies (sex bias; predominantly using a single sex), is also an important factor that can significantly affect underlying mechanisms and the DSS model study outcome [131,143,144,145]; sexual dimorphism is implicated in mechanisms of acute and chronic pain and inflammation [146], including liver drug-metabolising enzymes, at genetic, molecular, cellular, and whole-system levels in both rodents and humans. Increasing evidence shows that nociception and nociplasticity in visceral pain signal processing (involving neurons, glia, and immune cells in the peripheral and central nervous systems, and the communication of the gut microbiota with neural systems), including emotional pain perception, are sex-dependent [147].

#### 3.2.5. Gaps in Transparent Reporting of Genetic Factors

In DSS studies, the strain is most commonly reported (e.g., C57BL/6, abbreviated as B6), whereas the substrain is typically not specified (Table 6). In addition, the GEM line is only rarely reported correctly. For example, Table 6 shows two cases in which GEM lines (bold text) are presented in accordance with the nomenclature. Accurate and transparent reporting of both the exact GEM line and its breeder (origin) is essential to ensure valid and reproducible results. This level of detail allows researchers to use the same GEM animals or to verify their availability through established sources, such as The Jackson Laboratory, which provides commercially available mouse lines. A search of The Jackson Laboratory database indicates that the available GEM lines include **B6.129X1-*Trpv1^tm1Jul^*/J** and **B6;129P-*Trpa1^tm1Kykw^*/J** but not **B6.129P-*Trpa1^tm1Kykw^*/J.** The period between B6 and 129P (B6.129P) indicates a congenic line with a C57BL/6 genetic background carrying a targeted knockout mutation.The semicolon in “B6;129P” denotes a mixed genetic background.

Therefore, in the case of:

- **B6.129X1-*Trpv1^tm1Jul^*/J** line: The search results correspond to the entry reported in Table 6 (first row).
- **B6;129P-*Trpa1^tm1Kykw^*/J** line: The search result differs from the entry reported in Table 6 (third row), specifically in the use of a semicolon instead of a period/dot. Since Jackson Laboratory does not list a corresponding line with a period/dot (i.e., B6.129P), this suggests that the published entry may contain a typographical error.

Importantly, this seemingly minor difference in notation reflects a meaningful distinction in genetic background, which may have significant implications for reproducibility and interpretation of results.

#### 3.2.6. Traps and Tricks

Differences between substrains arise from the progressive accumulation of mutations (genetic drift) in genetic material. Most mutations are recessive and cannot be detected by visual observation. Therefore, the following is strongly recommended:

- Pay special attention to differences among substrains [151] and conduct the experiment on the same substrain.
- Pay attention to the genetic background of GEM and wild-type controls—use the same substrain in the study—mispairings between GEM and wild-type (WT) controls can lead to inaccurate and conflicting findings [152].
- Avoid GEM lines with a mixed genetic background because they lead to unexpected and non-reproducible results [153].
- Use both males and females in DSS model studies, unless justified otherwise [154].

A defined genetic background and knowledge about the origin of inbred laboratory animals are crucial for the validity and reproducibility of experimental studies. Therefore, studies should report all information regarding the animal genetic state according to the mouse and rat nomenclature available on https://www.informatics.jax.org/mgihome/nomen/strains.shtml (accessed on 10 March 2026) and LAG-R guidelines [155].

Importantly, the interpretation of a gene’s role can vary depending on the genetic background used (i.e., strain or substrain). Thus, generalising a gene’s role obtained from a single genetic background to another inbred strain or to humans can be misleading [156]. To get “generalised” insight into the gene’s role, various genetic backgrounds need to be tested. Interactions with different genetic backgrounds might lead to variability and even opposing outcomes, thereby providing better insight into the gene’s role in humans.

### 3.3. Microbiological Factors in DSS Colitis

**Figure 9:**
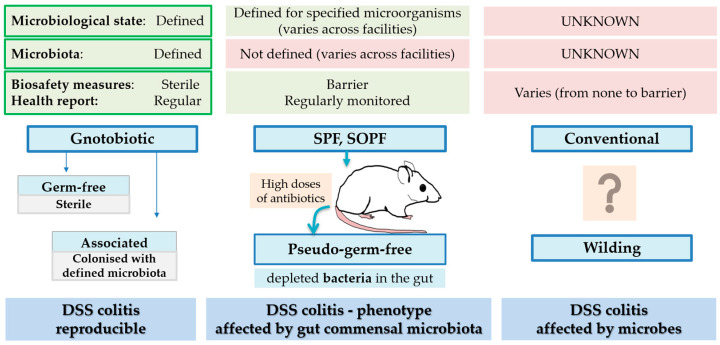
The microbiological status of animals affects the DSS colitis phenotype and reproductivity. Gnotobiotic animals are maintained in sterile conditions, and their microbiological state and microbiota are defined (green boxes with green borders), enabling microbiologically controlled DSS colitis experiments. SPF, SOPF, and PGF are typically maintained under barrier conditions, with regular monitoring of pathogenic or opportunistic microbes (so-called exclusion list, which differ across facilities) (green boxes). Health monitoring typically does not include the microbiota (the red box denotes an unknown or undefined state), leading to variability in DSS colitis phenotype. When the animals are not monitored for a specified list of microbes (conventional and wilding animals), the microbiological state of animals is not known (question mark).

The susceptibility and response to colitis development are significantly influenced by the microbiological (hygienic) status of the animals (i.e., gnotobiotic, SPF, conventional; Figure 9).

#### 3.3.1. Effect of Pathogenic and Opportunistic Microbes

Microorganisms can interfere with colitis, affecting its course, severity and immune signature. Pathogenic bacteria exhibit various modes of action within the organism and can significantly interfere with DSS colitis. Some microbes produce and secrete toxins that disrupt the mucosal barrier and promote injuries in the intestinal epithelial layer (*Bacteroides fragilis*, *Helicobacter hepaticus*, *Helicobacter cinaedi*, *Campylobacter jejuni*), while others influence the immune system [157]. For instance, *H. hepaticus* stimulates ILC3s to produce IL-22, which in turn promotes the development of colorectal cancer [158]. Some infections can reduce the severity of inflammation (*Strongyloides venezuelensis*) [159,160].

In most cases, infected mice do not exhibit clinical signs, and if animals are not routinely tested, the infection, although it affects the course or severity of colitis, remains undetected. An example is a study that reported a higher severity of colon inflammation, higher circulating inflammatory cytokine levels, and higher numbers of Th1 and Th17 cells in a murine colitis model in a conventional setting compared to mice maintained in an SPF facility. Routine health monitoring tests revealed that mice in conventional settings were infected with several pathogens (*Helicobacter hepaticus*, *Helicobacter typhlonius*, *Klebsiella oxytoca*, *Pasteurella pneumotropica biotype Heyl*, SFB—segmented filamentous bacteria), which were responsible for phenotype variability [161].

#### 3.3.2. Effect Without Microbes (Germ-Free)

**Figure 10:**
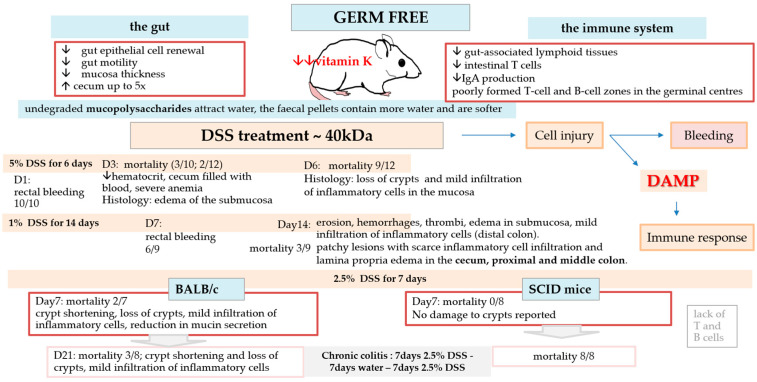
Germ-free mice and their clinical and histological response to DSS treatment. GF animals possess significant alterations in the intestine and the immune system. The most pronounced characteristic is the enlargement of the cecum (up to 5-fold×); the luminal content of the intestine is fluid, the faecal pellets contain more water and are softer (undegraded mucopolysaccharides attract water), the lamina propria is thin and almost without antibody-producing plasma cells, epithelial cell renewal is decreased, bowel motility is decreased (intestinal atonia) and lymph nodes are smaller. GF mice lack vitamin K, which is essential for blood clotting, as it is synthesised by the gut microbiota. All these characteristics affect sensitivity and response to DSS (40 kDa). Fluid content and decreased gut motility, the absence of microbiota, and decreased IgA concentration increase contact time for DSS and enable DSS to penetrate the mucosa in higher amounts, causing significant injuries that are manifested clinically as rectal bleeding. Animals develop prolonged prothrombin times and haemorrhages, very likely due to the limited amount of vitamin K and B provided in the food. Importantly, the immune system is underdeveloped and responds to DAMPs only (no PAMP/MAMPs in GF animals), which influences the type of immune cell infiltration and the intensity of inflammation (sterile inflammation). Histologically, changes are seen 3 days after DSS treatment, such as oedema of the submucosa (5% DSS), followed by hydropic degeneration of epithelial cells, which finally leads to loss of crypts (D6).

To eliminate the effect of microbes on colitis development, germ-free mice were used. The results were shocking (Figure 10). The typical DSS protocol for SPF mice (4–5% DSS 40 kDa for 3–6 days) resulted in rectal bleeding a day after DSS consumption and death 3 days later [162,163,164]. DSS caused significant injury to the colon of GF mice, even at low concentrations, such as 1% or 2.5% DSS [164,165], indicating that microbes play a crucial role in the development of colitis and inflammation.

The results obtained from germ-free animals are not directly translatable to humans, as they do not recapitulate the complexity of the microbiota present in humans. However, their contribution is invaluable when used in combination with studies in rodents with complex microbiota, for instance, in microbiota transfer, when examining the role of specific microbiota in DSS colitis susceptibility and variability [166].

#### 3.3.3. Effect of Antibiotic Treatment (Pseudo-Germ-Free)

In DSS studies, pseudo-germ-free animals (i.e., animals with a significantly reduced or altered gut microbiota, typically achieved through antibiotic treatment) are also used [163]. Unlike germ-free mice, which are completely devoid of microorganisms, pseudo-germ-free mice still harbour some residual microbes or have an altered microbial community [167]. It is important to take into consideration that antibiotics mostly eliminate bacteria, while other microbes (viruses, fungi, etc.) are still present. In addition, animals treated with high doses of antibiotics already have an established immune system.

Compared with working with germ-free animals in isolators, antibiotic treatment is less labour-intensive and can be initiated or discontinued at specific study points. The temporary depletion of the gut microbiota can be achieved using antibiotics, though complete elimination is challenging. Ampicillin is the most effective component but does not eradicate some microaerophilic Gram-positive bacteria, prompting the addition of vancomycin and neomycin. Metronidazole, which targets bacteria and flagellates, is sometimes included in drinking water formulations but should be used cautiously, preferably via gavage, to prevent issues related to drinking behaviour or toxicity. While administering antibiotics in water offers practical advantages, factors such as taste aversion, poor mixing, light exposure, and contamination can reduce effectiveness. Since antibiotics degrade over time, frequent water changes are necessary (for more information, see [167]).

As antibiotics and germ-free status influence the host differently, there are differences in the DSS colitis between studies of germ-free and pseudo-germ-free (antibiotic treated) mice (Table 7).

#### 3.3.4. Effect of Hygienic Measures

Today, laboratory rodents are maintained under highly controlled conditions, typically behind barrier systems that prevent microbial contamination. These colonies, referred to as “SPF”, are subject to comprehensive health monitoring programs designed to detect any microbial agents that could compromise the animals’ health (clinical infections), endanger personnel’s health (zoonoses), or impact research validity (subclinical infection). SPF animals have become the gold standard in animal research, including studies on DSS colitis. If any of the known bacterial or viral agents are identified (i.e., pathogens from the list of microorganisms recommended by the FELASA [168]; https://felasa.eu/working-groups; accessed on 10 March 2026), the usual course of action is depopulation of the affected colony to prevent further spread, highlighting the rigorous biosecurity measures.

However, these rigorous measures (rederivation, barrier protection conditions) have resulted in some adverse effects on research. For instance, many microorganisms crucial for modelling human disorders have been eliminated from animal facilities (which may lead to artificial reaction and the loss of animal model disease phenotypes, such as a lack of certain memory T cells) or have been found only in some facilities (which affects the reproducibility of animal models) [169].

#### 3.3.5. Effect of Wilding

To address this issue, researchers began using pet store or wild mice, which harbour microbiota that have evolved in a more complex, antigen-experienced environment (animals express a more human adult-like T cell profile). However, these mice also carry potentially harmful pathogens, which pose challenges such as disease outbreaks in animal facilities and 3R issues (GEM need to be housed in escape-proof closures, typically in facilities) [169].

#### 3.3.6. Effect of Gut Commensal Microbiota

Over the past two decades, research has intensely focused on the gut microbiota, including its role in the pathogenesis of IBD [170]. The gut microbiota has a complex relationship with the host. It is beneficial for the host due to its involvement in vital host physiological processes (e.g., digestive and metabolic functions), the maturation and activation of the immune system (responses), and the maintenance of the intestinal mucosal barrier, including protection against pathogen colonisation [171,172]. The gut microbiota and their metabolites play a crucial role in the neuroimmune system of the gut and, consequently, in the regulation of gut function, and can influence DSS colitis symptoms by modulating pain and gut dysmotility [5,63,65]. Conversely, the immune system, neuronal factors, and neuropeptides influence the composition of the microbiota.

However, gut microbiota differ among the same strains of animals from different vendors [173,174], facilities [175] or even in mice of the same strain housed in separate units within a commercial breeding facility [176], which causes DSS colitis variability. Li et al. reported a significant difference in DSS colitis severity across three separate orders of C57BL/6J mice from the same commercial vendor, despite mice being of the same substrain, sex, microbiological state (SPF), and age at each shipment. Ultimately, it was discovered that each shipment of mice had a distinct gut microbiota composition upon arrival, which was responsible for differences in the DSS colitis phenotype [177].

Differences in the gut microbiota can occur already after three generations of separate breeding (due to differences in barrier husbandry practices in both the parental and filial generations) [178]. Maternal transmission was found to be a major contributor to shaping the composition of the gut microbiota [179]. In-house-bred animals, particularly transgenic or knockout mice, are at a high risk of diversity of gut commensal microbiota and its impact on DSS colitis and conclusions. For instance, Brinkman et al. found differences in DSS colitis severity between knockout mice (caspase-3-deficient) and their WT counterparts, and attributed these differences to caspase-3 deletion. However, further studies using the co-housing strategy have shown that there was no difference between knockout (KO) and WT mice when the effect of gut microbiota (*Prevotella* spp.) was excluded (no genotype effect) [180].

The gut microbiota comprises approximately 10^10^–10^14^ microorganisms (i.e., bacteria, fungi, protozoa, archaea, yeast, viruses). Monitoring a whole gut microbiome [181] would be time- and cost-consuming. In addition, new microbes are identified every year. Thus, how should we control or monitor the effect of microbiota in the colitis model? It was recommended that commensal bacteria with an identified impact on research results become part of health monitoring tests [182] and an essential component of reporting in publications, particularly because these bacteria vary among vendors, facilities, and laboratories. The inconsistent presence of anti- or proinflammatory bacteria (Table 8) in contemporary rodent facilities affects the reproducibility of animal models, including the DSS model. It is important to keep in mind that most research currently focuses on gut bacteria, which is why recommendations are made only about bacteria.

#### 3.3.7. Traps and Tricks

Microorganisms, including the gut microbiota, have a profound influence on IBD aetiology and pathophysiology, as well as on phenotype variability in DSS colitis [166,183]. To reduce microbiome-related bias, the following is recommended:

- Monitor and standardise microbiome-related variables to improve the reproducibility and interpretation of DSS colitis experiments (bedding, nesting material, diet, enrichment, hygienic measures, health monitoring tests, etc., avoid variation within the study).
- Allow experimental design to test for the cage factor (particularly in the case of microisolators or individually ventilated cages (IVC) housing).
- Co-house transgenic/knockout mice with WT mice to balance microbiota.
- When using in-house-bred mice, account for all factors that could affect the gut microbiome and control them, particularly microbiota composition, litter effects, and genetic background (see also the section on genetic factors).

Numerous factors can affect microbiota composition. If animals are housed differently, even small changes can shift gut microbes over time. Examples include bedding type (paper vs. corn cob) and diet, which can alter the microbiota composition over weeks [184], as well as alterations in bedding during food restrictions [185].

## 4. Translational Relevance and Limitations of the DSS Model

The DSS model represents a model in which epithelial injury, microbes, and host genetics converge to drive intestinal inflammation. Although DSS-induced colitis reproduces many pathological and clinical features of UC, it differs from human UC in its initiating mechanisms and immune complexity. The DSS model does not reliably reproduce transmural inflammation or granuloma formation, limiting its utility as a CD model. Similarities between the DSS model and CD and UC are shown in Table 9.

Despite these limitations, the DSS model remains a valuable model for investigating epithelial barrier function, immune responses, and inflammatory signalling pathways. However, the commonly cited notion that an ideal IBD model should closely mimic clinical disease and reliably predict therapeutic efficacy oversimplifies both the complexity of IBD and the limitations of the DSS model. Human IBD encompasses a spectrum of phenotypes requiring personalised therapeutic approaches, whereas DSS-induced colitis is largely driven by acute injury and may resolve spontaneously after a single cycle. Consequently, its use as a universal model for testing therapeutic agents is limited and may lead to misleading conclusions, particularly when based primarily on clinical scores rather than comprehensive histological and molecular analyses.

### Recommendations and Future Directions

As shown, disease severity and outcomes in the DSS model are strongly influenced by experimental variables, including genetic background (strain and substrain differences), microbiological status, DSS protocol, and the timing of assessment. Differences in housing conditions, pathogen exposure, and microbial composition can lead to substantial variability in disease severity and immune responses. This represents an important experimental confounder and emphasises the need for strict microbiological monitoring and reporting or, alternatively, deliberate microbiota manipulation in experimental design. In addition, genetic background is often an underappreciated determinant of DSS colitis outcomes. Strain-dependent differences may affect not only susceptibility but also immune response and underlying mechanisms. These may have important implications for translational research. The widespread reliance on a single inbred strain (most commonly C57BL/6) may limit the generalizability of results and fail to capture the genetic heterogeneity observed in human IBD. Future DSS studies should incorporate multiple genetic backgrounds and strains. Such an approach would better reflect the polygenic nature of IBD and improve the robustness and translational relevance of findings. A deeper understanding of how genetic, microbial, and environmental factors interact in DSS-induced colitis will be essential to improve its predictive value and align experimental findings more closely with human IBD pathophysiology and treatment responses.

## 5. Conclusions

As technology advances, our opportunities to study complex diseases have expanded significantly, leading to substantial progress in our understanding of IBD. Today, the investigation focuses on the cellular and molecular mechanisms underlying IBD pathogenesis. The research examines the complex interplay among molecular factors from various tissues, organs, and systems that are interconnected in the maintenance and disruption of homeostatic physiological processes. The deeper we delve, the more complex and diverse mechanisms at the various levels (cellular, tissue, organ, system) of the organism we obtain. When looking at the broader picture—organisms as a whole—we can see numerous similarities and countless possibilities for how organisms maintain homeostasis or cure/repair pathology in the body. The easiest way to seek the scientific answer is to reflect on the results, understand the animal models used, and design experiments in accordance with state-of-the-art guidelines and knowledge.

The review comprehensively illustrates the complexity of the DSS colitis model, showing that, as in humans, genetic factors, microbes, gut microbiota, environmental influences, and the immune system all play roles in the development and modulation of colitis in an animal DSS model. DSS colitis depends heavily on leukocyte recruitment responses (which vary with germ-free/SPF/microbiota/infection status) and on the production of inflammatory mediators (which vary with genetic background/strain/age/immune status) (Figure 8), all of which contribute to variability in tissue damage, immune responses and molecular signature. In addition, the review synthesises the effects of DSS colitis on other organs (the liver and brain) and their metabolism and molecular responses, which may, in turn, influence the colitis phenotype, drug response, and the interpretation of results. This results in various morphological, cellular, and molecular forms of colitis, leading to variability in the DSS colitis phenotype, nonreproducibility, and even conflicting study outcomes.

The DSS model has already yielded controversial results among laboratories and research groups. For instance, Zaki et al. reported that NLRP3-deficient mice were more prone to DSS-induced colitis compared to wild-type mice, showing increased mortality and morbidity [186]. Conversely, Bauer et al. reported that NLRP3-deficient mice showed a milder colitis phenotype than wild-type mice, and reduced levels of proinflammatory cytokines in their colonic tissue following DSS treatment [187]. In such a situation, we would usually ask ourselves whose study is right. However, due to the multifactorial nature of the DSS colitis model, the answer is not simple. We should ask ourselves which factor in those two studies was responsible for the diverse study outcomes. As shown in the review, molecular factors may play dual roles in DSS colitis or can be differently involved in underlying mechanisms due to the plasticity and complexity of colitis. This shows that the interpretation of the results is more complex and requires a consideration of numerous factors influencing DSS colitis, and that the results should be interpreted within a stage- and context-dependent framework of DSS colitis.

Thus, this review not only identifies the numerous factors that can confound DSS study outcomes but also provides concrete examples and mechanistic explanations of how these factors influence results, with the intention to encourage researchers to report all factors/details and events that are taking place during the experiment and in a mouse. In this way, we can simultaneously gather additional information and insights about the complex organism, which is also in accordance with the rational use of animals (to obtain more information with fewer animals, 3R), particularly because, in the future, AI tools and mathematical models will enable us to analyse big data more complexly.

The conflicting results in DSS colitis studies thus show that confounding factors can influence the disease, either directly or indirectly. Differences between studies arise from a complex interplay between known (measured and controlled) factors and unknown (uncontrolled) factors. When we have complex, multifactorial diseases like IBD or DSS colitis and tools capable of processing large amounts of data, it is essential to report details that have been shown to impact the disease model. New factors may be identified each year, expanding the list of known influences. While this can seem daunting, it helps reveal the intricate mechanisms behind how various factors interact and affect outcomes. This underscores the importance of thorough reporting of all variables that affect the model, beyond what ARRIVE guidelines require.

We encourage researchers to report all factors and circumstances that may affect the model and the study’s outcome, including errors and potential hurdles encountered during the study. All this information is helpful and useful (see CIRS Critical Incident Reporting System—Laboratory Animal Science: https://www.cirs-las.org/home).

A heterogeneity of factors across studies can contribute to a better understanding of the underlying mechanisms, but only if all details are controlled, monitored and reported.

To provide a user-friendly framework, information is gathered in a table or checklist at the end, serving as a practical guide for improving the execution and/or reporting of future DSS studies (Table 10).

## Figures and Tables

**Table 1:** Pain-related molecular markers and sensitivity to mechanical and thermal stimuli in mice with acute DSS colitis and after colitis resolution (without any mechanical or chemical stimuli to evoke visceral hyperalgesia).

Tissue	D7–D9 Acute DSS Colitis (Acute Inflammatory Pain)	D42–D49 (Post-Inflammatory Hypersensitivity)
Colon	D9: ↑ *Ifn-γ*, ↑ *Il-1β*, ↑ *Tnf-α*,↑ *Il-10*; ↑ Trpv1 [68,69]	D49: recovered no changes from normal [68,69]
	D7: ↑ *Tnf-α*, ↑ *Il-6*, ↑ *G-csf* ~Trpv1; ↑ SP, ↑ Cgrp [70]	D42: ~*Tnf-α*, *Il-6*, *G-csf*; ↑ Trpv1; ↑ SP, ~Cgrp [70]
DRG	D7: T10-L1, L6-S1: ~*Trpv1*	D42: T10-L1, L6-S1: ↑ *Trpv1* [70]
	D8: L6-S1: ↑ *Trpa1*, ~*Trpv1*, ~*Trpv4* TG: ~Trpy1 [72] D8: lumbosacral: ↑ pp42/44 Mapk, ↑ c-Fos [73]	na
brain	D8: ↑ cFos in thalamus, hypothalamus, amygdala, prefrontal cortex [73]	na
Sensitivity to: Mechanical stimuli (von Frey test)	↑ abdominal skin (referred pain) [72,73] ↑ plantar side of hind paws [73] ↑ facial skin [72]	↑ abdominal skin [68,69]
Thermal stimuli	↑ plantar side of hind paws [73]	na

Legend: Cgrp—calcitonin gene-related peptide; D9—day 9; DRG—dorsal root ganglia; G-csf—granulocyte colony-stimulating factor; Ifn-γ—interferon gamma; L6-S1—lumbosacral; pp42/44 Mapk—phosphorylated p42/44 Mapk; SP—substance P; T10-L1—thoracolumbar; TG—trigeminal ganglion; Tnf-α—tumour necrosis factor alpha; Trpv1—transient receptor potential vanilloid 1; Trpa1—transient receptor potential ankyrin 1; ↑—denotes significantly increased; ~—denotes no significant difference; na—not analysed.

**Table 2:** Example of alterations in drug-metabolising enzymes in liver of animals with DSS colitis.

Parameters Measured	DSS vs. Control		Ref.
Liver mRNA (RT-qPCR) normalisation (*Hprt*)	D7: ↑ *Il-1β* ↓ *Cyp1a1*, ↓ *Cyp1a2*, ~*Cyp2b10*, ↓ *Cyp2c38*, ↑ *Cyp3a11*, ↑ *Cyp3a13*		[115]
Enzyme activity assays	D7: ~ Cyp1a1/2, ↑ Cyp2b, ↑ Cyp2c, ↑ Cyo3a		
Blood plasma levels (pg/mL)	D7: ~ Ifn-γ, ~ Il-6, ~ Il-10		
Intestinal epithelium and liver mRNA	D7 (colon): ↑ *Il-1β*, ↑ *Il-6*, ↑ *Tnf-α* (colon) D7 (liver): ↓ *Cyp1a2*, ↓ *Cyp2b10*, ↓ *Cyp2c29*, ↓ *Cyp3a11* D7 (proximal part small intestine): ↓ *Cyp1a1*, ↓ *Cyp2b10*, ↓ *Cyp2c29*, ↓ *Cyp3a11*		[113]
Proteins (immunoblot)	D7 (small intestinal microsomes): ~ Cyp1a, ↓ Cyp2b, ↓ Cyp2c, ~ Cyp3a		
Liver mRNA (RT-qPCR) normalisation (*β-actin*)	D10: liver histology: bp; blood plasma: ~ALT, ~AST D10: ↑ *Il-1β*, ↑ *Il-6*, ↑ *Tnf-α* D10: ↓ *Cyp1a2*, ↓ *Cyp2c29*, ↓ *Cyp2d9*, ↓ *Cyp2e1*, ↓ *Cyp3a11*		[108]
Protein expression (Western blot) normalisation (Gapdh)	D10: ↓ Cyp1a2, ↓ Cyp2c, ↓ Cyp2d, ↓ Cyp2e, ↓ Cyp3a D10 enzyme activity: ↓ Cyp3a		
Liver mRNA (RT-qPCR) normalisation (β-actin)	D4: nd LPS, ↑ *Il-1β*, ~ *Il-6*, ~ *Tnf-α* D7: nd LPS, ↑ *Il-1β*, ~ *Il-6*, ↑ *Tnf-α* D10: ↑ LPS, ↑ *Il-1β*, ↑ Il-6, ↑ *Tnf-α* D20: ↑ LPS, ↑ *Il-1β*, ~ *Il-6*, ↑ *Tnf-α* D50: ↑ LPS, ~ *Il-1β*, ~ *Il-6*, ~*Tnf-α*	Relative weight of liver bp, histology bp	[109]
(A) Liver mRNA (RT-qPCR) normalisation (β-actin) (B) Protein expression (Western blot) Cyp3a	(A) D4: ~ *Cyp1a2*, ~ *Cyp2c29*, ~ *Cyp2d9*, ~ *Cyp2e1*, ~ *Cyp3a11* D7: ↓ *Cyp1a2*, ~ *Cyp2c29*, ~ *Cyp2d9*, ~ *Cyp2e1*, ↓ *Cyp3a11* D10: ↓ *Cyp1a2*, ↓ *Cyp2c29*, ↓ *Cyp2d9*, ↓ *Cyp2e1*, ↓ *Cyp3a11* D20: ~ *Cyp1a2*, ↓ *Cyp2c29*, ↓ *Cyp2d9*, ~ *Cyp2e1*, ↓ *Cyp3a11* D50: ~ *Cyp1a2*, ~ *Cyp2c29*, ↓ *Cyp2d9*, ~ *Cyp2e1*, ~ *Cyp3a11*	(B) D4: ~ Cyp3a D7: ↓ Cyp3a D10: ↓ Cyp3a D20: ↓ Cyp3a D50: ~ Cyp3a	

BALB/c females, 2 months; 2.5% DSS (36–50 kDa; Biomedicals) for 1 week; refreshed every day and autoclaved (2x repeated experiments); SPF settings, free of FELASA standard health monitoring list of pathogens; Euth: isoflurane, cervical dislocation; blood (EDTA) [115]. C57BL/6 males, 8–12 weeks; 2.5% DSS for 7 days; blood samples from tail vein 10, 30 min, 1, 2, 4, 10 h after drug treatment [113]. ICR males, 7 weeks; 3.5% DSS (36–50 kDa Biomedicalsfor) 10 days; Euth: diethyl ether and blood from abdominal cava using heparin; colon, liver, spleen [108]. ICR males, 7 weeks; 3.5% DSS (36–50 kDa Biomedicals) for 10 days + 40 days water; d4, d7, d10, d20, d50 blood Hb, plasma LPS [109]. Legend: ALT—Alanine Aminotransferase; AST—Aspartate Aminotransferase; Cyp1a—cytochrome P450 family 1a; D4—day 4; Il6—interleukin-6; Ifn-γ—interferon gamma; LPS—bacterial lipopolysaccharide; nd—not detected; Tnf-α—tumour necrosis factor alpha; ↑—denotes significant increase; ↓—denotes significant decrease; ~—denotes no significant difference.

**Table 3:** Differences in the anatomical site, severity of inflammation and the incidence of erosions in DSS colitis among mouse strains (adapted from [131]).

	CECUM		COLON			
Mouse STRAIN	E		E	Proximal	Middle	Distal
C3H/HeJBir	83%	++/+++	83%	−/+	+/++	+++
C3H/HeJ	77%	++	88%	−/+	+/++	+++
NOD/LtJ	75%	++/+++	75%	−	++	++/+++
NOD/LtSz-*Prkdc^scid^/Prkdc^scid^*	69%	++	56%	−/+	+/++	++/+++
DBA/2J	42%	+/++	0%	−	+	+/++
C57BL/6J	31%	+	69%	−/+	++	++/+++
NON/LtJ	17%	−/+	25%	−	+	+
NON.NOD-*H2^g7^*	0%	+	14%	−	+/++	+/++
129/SvPas	0%	−	75%	−	+/++	++

Legend: E—incidence of animals with erosion; − no hystological lesion (normal mucosa); −/+ scarce inflammation; + mild colitis; ++ moderate colitis; +++ severe colitis. DSS protocol: 3.5% DSS (36–45 kDa) for 5 days, D21 euthanasia (CO_2_) [131].

**Table 4:** Examples of spontaneous mutations among C57BL/6 substrains that can affect the DSS model.

SUBSTRAIN	Vendor	*Nnt*	*Snca*	*Mmrn1*	*Crb1^rd8^*
C57BL/6**J**	**J**ackson Lab	yes	no	no	no
C57BL/6**JCrl**	**C**ha**rl**es River	yes	no	no	no
C57BL/6**ByJ**	**J**ackson Lab	no	no	no	no
C57BL/6**JOlaHsd**	Envigo (Harlan)	no	yes	yes	no
C57BL/6**JRccHsd**	Envigo (Harlan)	no	no	no	no
C57BL/6**NCrl**	**C**ha**rl**es River	no	no	no	yes
C57BL/6**NHsd**	Envigo (Harlan)	no	no	no	yes

Affected gene (mutant allele): *Nnt*—nicotinamide nucleotide transhydrogenase; *Snca*—locus encoding α-synuclein and *Mmrn1*—multimerin-1; *Crb1^rd8^*—crumbs like 1, retinal degeneration mutation. Red letters after the strain designation indicate the substrain. These letters are laboratory’s registration code where the mouse strain is created and maintained. For instance, “J” is the registration code for Jackson Laboratory, “Crl” for Charles River, and C57BL/6JCrl denotes a substrain created at Jackson Lab and bred at Charles River.

**Table 5:** Behavioural and neurochemical differences in substantia nigra between C57BL/6J and C57BL/6JOlaHsd substrains [141].

C57BL/6	J vs. C57BL/6JOlaHsd
Locomotor activity	↑ 33%
Thigmotaxis	↑ 38%
Endurance (Rotarod test)	↓ 33%
Tyrosine hydrolase-positive neurons	~34% vs. 40%
SN GFAP-jr cells	↑ 2-fold x

Legend: ↑—denotes increase in C57BL/6J in comparison to C57BL/6JOlaHsd; ↓—denotes decrease;

**Table 6:** Examples of reporting about genetic factors in DSS studies evaluating the role of a deleted gene in a DSS colitis model (strain, strain origin, genetic background, sex, age).

GEM	Background (Breeder)	Sex, Age, N	MW of DSS	DSS Treatment	End	S	Ref.
TRPV1^−/−^	**B6.129X1-*Trpv1^tm1Jul^*/J** (Jax) C57BL/6 (Crl)	6 wks; N = 6–12	nr	2.5% for 7 d	D7 D42	~	[70]
TRPV1^−/−^ TRPA1^−/−^	C57BL/6 (Japan SLC)	Male N = 8–10	35–50 kDa	2% for 7 d	D7	↓ ↓	[148]
TRPA1^−/−^	**B6.129P-*Trpa1^tm1Kykw^*/J** (Jax) C57BL/6 (Envigo)	Male, 8 wks N = 6–8	36–50 kDa	2% for 7 d	D8	~	[72]
TRPA1^−/−^	C57BL/6 University of Florence	8–10 wk N = 14–15	nr	2% for 7 d	D3 D7 D10	~ ~ ↑	[149]
TRPA1^−/−^ CGRP^−/−^ SP^−/−^	** Glaxo Smith Kline, UK Harvard, Boston, USA University Bonn, Germany	N = 6–13	36–50 kDa	2% for 7 d	D8	↓ ↑ ↓	[150]

Legend: D7—day 7; GEM—genetically engineered mouse; MW—molecular weight; N—number of animals per group; nr—not reported; S—susceptibility to DSS colitis in comparison to WT or control group. ** breeding pairs were donated; ↑—denotes increased susceptibility to DSS colitis; ↓—denotes decreased susceptibility.

**Table 7:** Changes in molecular parameters in DSS colitis among GF, PGF, and SPF mice (results summarised from [163]).

Parameters Measured	SPF Mice	PGF Mice	GF Mice
Colonic MPO activity	~ MPO (2% DSS) ~ MPO (4% DSS)	↑ MPO (2%) ~ MPO (4%)	↓#MPO (2%) ↓#MPO (4%)
Colonic AP activity	↑ AP (2%) ↑ AP (4%)	~ AP (2%) ↑# AP (4%)	↑# AP (2%) ↑# AP (4%)
Western blot Colonic proteins	↑ claudin-4, ↑ Pcna, ↑ p-STAT3, ~ZO-1, occludin, claudin-2, ~ cytokeratin 5/8	↓# claudin-2, ↓# claudin-4, ↓# Pcna, ↓ ZO-1 (4%), ↑ p-STAT3, ~ occludin, ~ cytokeratin 5/8,	↓# claudin-2, ↓# claudin-4, ↓# Pcna, ↓# p-STAT3, ↓# ZO-1, ↓# occludin (4%), ↓# cytokeratin 5/8
Colonic mRNA	↑ *Il-22* (4%), ↑ *Il-10* (2%), ↑ *Foxp3* (2%), *~ Il-27*, *Kgf*, *cyclin D1*, *Myc*, *~VEGF*	↓# *Foxp3* (2%), ↑# *Foxp3* (4%), ↑# *Kgf* (4%), ↑# *VEGF* (4%), ↑# *Il-10* (4%), *~ Il-22*, *Il-27*, *cyclin D1*, *Myc*	↓# *Il-22* (4%), ↑# *Il-27* (4%), ↓# *cyclin D1* (4%), ↓# *Il-10* (2%), ↓# *Foxp3* (2%), *~ Kgf*, *Myc*, *VEGF*
Colonic mRNA	↓ *occludin* *~Muc3*, *ZO-1*, *Tff3*, *Reg3γ*	↑ *Reg3γ* (4%), ↑# *Tff3,* ↑# *occludin* (0%, 2%), ↑# *ZO-1* (2%), *~ Muc3*	↑# *Reg3γ* (2%), *~ Muc3*, *Tff3*, *ZO-1*, ↓# *occludin* (0%)
Colonic mRNA	↑ *Ifn-γ*, ↑ *Il-1β* (2%), ↑ *S100A8 (2%)*, *~ Il-17, Tnf-α*,	↑ *Ifn-γ* (4%), ↓# *Il-1β* (2%), *~ Il-17, Tnf-α, S100A8*	↑# *Il-17,* ↑# *Tnf-α* (4%), ↓# *Ifn-γ,* ↓# *Il-1β* (2%), ↓# *S100A8* (2%)
ELISA MLNC	↑ Tnf-α, ↑ Ifn-γ, ↑ Il-6, ↑ Il-17 (4%)	↑ Tnf-α, ↑# Ifn-γ (4%), ↑# Il-6 (2%), ↓# Il-17 (4%)	↓# Tnf-α, ↓# Ifn-γ, ↓# Il-6 (4%), ↓# Il-17 (4%)
ELISA (splenocytes)	↓ Tnf-α (4%), ~ Ifn-γ, ↑ Il-6 (2%), ↑ Il-17	↓# Tnf-α (0%, 2%), ~ Ifn-γ, ~ Il-6, ↓# Il-17 (4%)	↓# Tnf-α, ↓# Ifn-γ, ↓# Il-6, ↓# Il-17,

Control (0% DSS) and DSS colitis induced by 2% or 4% DSS (36–50 kDa) for 7 days, euthanasia on d7 (n = 8 mice, 4 females and 4 males; GF mice: NMRI, Karolinska Institutet, Sweden; SPF mice: NMRI, Janvier-Labs, France; PGF mice: SPF mice NMRI, Janvier-Labs, treated with antibiotic cocktail (ampicillin 1 g/L, neomycin 1 g/L, metronidazole 0.25 g/L, and vancomycin 0.5 g/L). Legend: AP—alkaline phosphatase; Foxp3—Forkhead box P3; GF—germ-free; Il6—interleukin-6; Ifn-γ—interferon gamma; Kgf—keratinocyte growth factor; MLNC—mesenteric lymph node cells; MPO—myeloperoxidase; Muc3—mucin 3; Myc—MYC proto-oncogene; PGF—pseudo-germ-free; Pcna—Proliferating Cell Nuclear Antigen; p-STAT3—Signal Transducer and Activator of Transcription 3; Reg3γ—Regenerating islet-derived protein 3 gamma; S100A8—calcium binding protein A8; SPF—specific pathogen-free; Tnf-α—tumour necrosis factor alpha; Tff3—trefoil factor 3; VEGF—Vascular Endothelial Growth Factor; ZO-1—zonula occludens-1. ↑—denotes significant increase vs control (0% DSS); ↓—denotes significant decrease vs control (0% DSS); ↑#—denotes significant increase vs SPF; ↓#—denotes significant decrease vs SPF.

**Table 8:** Examples of commensal gut bacteria with documented impact on colitis models (modified from [182] and updated by [166]).

Commensal Gut Bacteria	Impact on Colitis
*Alistipes okayasuensis*	Increased severity; proinflammatory
*Akkermansia muciniphila*	Decreased severity; anti-inflammatory
*Bifidobacterium* spp.	Decreased severity; anti-inflammatory
*Bacteroides fragilis*	Proinflammatory
*Bacteroides vulgatus*	Proinflammatory
*Duncaniella muricolitica*	Increased severity; proinflammatory
*Faecalibacterium prausnitzii*	Decreased severity; anti-inflammatory
*Prevotella* spp. *P. copri*	Increased severity; proinflammatory
*Segmented filamentous bacteria* *(SFB or Candidatus Savagella)*	Increased severity; proinflammatory

**Table 9:** Similarities and differences between DSS colitis, CD and UC.

Category	DSS Colitis	UC	CD
Disease trigger	Chemical (DSS-induced mucus and epithelial disruption)	Multifactorial (genetics, microbiota, environment)	Multifactorial (genetics, microbiota, environment)
Primary mechanism	Barrier disruption → microbial translocation → immune activation	Barrier dysfunction + dysregulated immune response	Dysregulated immune response + barrier defects
Microbiological influence	Strong (housing conditions, pathogens, microbiota composition affect severity)	Dysbiosis	Dysbiosis
Genetic influence (strain-specific)	Strain dependence; differences in susceptibility, immune response, and chronicity	Polygenic	Polygenic
Localization	DSS protocol and strain dependence; colon (distal predominance)	Colon (continuous)	Entire GI tract (segmental)
Lesion pattern	Diffuse or patchy (protocol-dependent)	Continuous	Skip lesions
Clinical features (acute)	Weight loss, alteration of faeces consistency, bleeding, abdominal pain	Diarrhea, bleeding, abdominal pain	Diarrhea, abdominal pain
Histopathology	Crypt loss, mucin depletion, architectural distortion, erosion, neutrophil, lymphocyte infiltration	Cryptitis, crypt abscesses, mucosal inflammation	Focal inflammation, granulomas
Depth of inflammation	Mostly mucosal/submucosal (UC-like); protocol dependant	Mucosal/submucosal	Transmural
Systemic effects	May affect liver, brain (gut–organ axes)	Colon, abdominal pain	Extraintestinal manifestations

Legend: CD—Crohn’s disease; GI—gastrointestinal tract; UC—ulcerative colitis.

**Table 10:** Checklist to help monitor and report factors affecting DSS colitis outcomes.

Parameters to Report	Description and Comments
	Genetic factors section
Strain, substrain	Strain, substrain (nomenclature), source of origin
Genotype—GEM	Mutation, mode of creation, background substrain (use nomenclature); source of origin
Origin or breeding	Provide the origin or source breeder; when in-house breeding is used, provide information on the generation of breeding or backcrossing
Age	Age at which the experiment was initiated and timeline for experiments performed
Sex	Use both sexes and explain the statistical method (pooled or stratified analysis)
	**Microbiological factors section**
Microbiological status	Gnotobiotic, SPF, SOPF, conventional; MUST provide results of the health monitoring report list (varies across facilities)
Gut microbiota	Provide monitoring results of gut commensal bacteria from Table 8 Report hygienic (biosafety and biosecurity) measures and results of microbiota tests. Report all information about the diet, bedding, nesting material, and enrichment, stated below, and avoid dietary variation within the study
Diet	Type, supplier and catalogue number of commercially available diets; pretreatment (i.e., sterilisation/autoclave, gamma irradiation)
Water	Type, pretreatment (i.e., sterilisation, acidification, prefiltration, etc.)
Bedding and nesting material	Type, supplier, and catalogue number of commercially available certified material and pretreatment (i.e., sterilisation/autoclave, gamma irradiation, disinfection—provide disinfectant)
Enrichment	Include description about enrichment items, supplier, pretreatment (i.e., sterilisation/autoclave, gamma irradiation, disinfection—provide disinfectant)
Type of housing	IVC system, open cages, microisolators
Animal housing	Single or group, number or animals per cage, cage size (floor area); co-housing
Light	Light–dark cycle, lights on/lights off; light intensity
Temperature, humidity	Range of temperature and relative humidity during the experiment
Acclimatisation	Duration of acclimatisation and habituation
	**Experimental design**
Experimental design	Define the number of animals per group Use a completely randomised or randomised block design to assign animals to groups [188] Clearly report whether blinding and randomisation were used during housing, treatments, measurements, necropsy and sampling [189] Define the experimental unit and avoid pseudoreplication (i.e., DSS solution in drinking bottles in group-housed animals) [190]
Group allocation	Method of allocation to the groups in regard to litter effect, microbiota or genetic background
Groups	Due to numerous factors influencing the model, control groups, positive and negative, are recommended, particularly when the model is used to test an agent or testing strategy
DSS	Molecular weight, concentration, preparation, water type, duration, frequency Rectal bleeding is a serious symptom in DSS studies; it requires prompt removal of DSS to prevent animal death; simple monitoring steps, such as daily checks and changing white cellulose towels in cages, can help prevent severe outcomes
Treatments	Compound, mode of delivery, type of vehicle, amount (volume), time of the day of administration and frequency of treatments
Behavioural tests	Randomization of the groups, line orders of execution—assigned to experimental setting, Blinded to genotype, drug treatment Sex of the operator, number of operators performing the tests Duration of the test, acclimatisation to object, cleaning method (to remove olfactory cues) Time of the day and time window, when tests was performed during the whole study
Euthanasia	The method and timing of euthanasia can influence liver metabolism, gut–brain axis signalling, and other molecular parameters in the gut
Sampling	State time of the day of experimental procedures and sampling, and time window of sample collection Perform all procedures, sampling, and euthanasia at the same time each day and within a narrow time window across all groups to minimise circadian effects on molecular parameters. When measuring colon length, remove the entire colon with the rectum and anus from the animal Note that mice lack Paneth cells in their colon (unlike humans)

## Data Availability

No new data were created or analyzed in this study. Data sharing is not applicable to this article.
